# Prevalence of Dyslipidemia and Lipid Goal Attainment in Statin-Treated Subjects From 3 Data Sources: A Retrospective Analysis

**DOI:** 10.1161/JAHA.112.001800

**Published:** 2012-12-19

**Authors:** Peter H. Jones, Radhika Nair, Kamlesh M. Thakker

**Affiliations:** 1 Baylor College of MedicineHouston, TX; 2 Abbott, Abbott Park, IL

**Keywords:** coronary heart disease, dyslipidemia, low-density lipoprotein cholesterol, non–high-density lipoprotein cholesterol, statins

## Abstract

**Background:**

Evidence-based randomized clinical trials have shown significant benefit of statin treatment with regard to cardiovascular disease. In anticipation of the National Cholesterol Education Program Adult Treatment Panel IV guidelines, we wanted to assess the current state of lipid goal attainment in the high-risk secondary prevention population in the United States. The objectives of the study were to estimate the proportion of high-risk patients treated with statin monotherapy who achieved Adult Treatment Panel III–recommended low-density lipoprotein cholesterol (LDL-C) goals (<100 mg/dL; optional <70 mg/dL) as well as non–high-density lipoprotein cholesterol goals (<130 mg/dL; optional <100 mg/dL).

**Methods and Results:**

This is a cross-sectional, retrospective study of 3 data sources: electronic medical records (2003–September 2010), administrative claims data (2003–2010), and National Health and Nutrition Examination Survey data (2007–2008). High-risk patients (≥18 years of age) were defined as those with a history of coronary heart disease or coronary heart disease risk equivalent who had the latest complete lipid panel measurement and had been treated with statin monotherapy for >90 days at the time of the lipid panel. Cardiovascular disease, coronary heart disease, and coronary heart disease risk equivalents were defined on the basis of availability, specific to each data source. Across the 3 data sources, 20% to 26% of high-risk patients treated with statin monotherapy for >90 days had LDL-C <70 mg/dL, and 67% to 77% had LDL-C <100 mg/dL. The percentages of those attaining both LDL-C goals and non–high-density lipoprotein cholesterol goals were quantitatively smaller (13.5% to 19.0% and 46% to 70%).

**Conclusions:**

Across the 3 data sources, there was consistency in the proportion of high-risk patients treated with statin monotherapy who were at LDL-C goal. A significant number of these statin-treated patients had additional dyslipidemias.

## Introduction

Treatment guidelines for reducing cardiovascular risk focus on lowering low-density lipoprotein cholesterol (LDL-C) on the basis of extensive evidence from primary and secondary prevention trials with statins.^1^ Treatment goals for LDL-C, as specified by the National Cholesterol Education Program (NCEP) Adult Treatment Panel (ATP) III, are risk stratified: <100 mg/dL for high-risk patients (coronary heart disease [CHD] or CHD risk equivalents, 10-year risk >20%), <130 mg/dL for moderate-risk patients (≥2 risk factors, 10-year risk 10% to 20%), and <160 mg/dL for low-risk patients (0 to 1 risk factors). CHD risk assessment is generally evaluated with the Framingham scoring system, which takes into account cigarette smoking, treated or untreated systolic hypertension, sex, age, total cholesterol level, and high-density lipoprotein cholesterol (HDL-C) level.^2^ The 2004 update to the NCEP ATP III added an optional LDL-C goal of <70 mg/dL for very high-risk patients (established cardiovascular disease [CVD] with multiple major risk factors), which also has been endorsed by the American Heart Association, the American College of Cardiology, and the American Diabetes Association.^2–5^

Although statins are excellent at reducing LDL-C and remain the mainstay of lipid-modifying therapies, patients optimally treated with statins continue to have cardiovascular events.^6^ One potential reason for this might be suboptimal non–HDL-C levels. The NCEP ATP III recommends non–HDL-C as a secondary target in patients with elevated triglycerides (TG) (≥200 mg/dL) after the LDL-C goal has been achieved.^2^

There is some confirmation, based on recent evaluations and reviews, that non–HDL-C does have an impact on cardiovascular outcomes.^6–9^ Additionally, Boekholdt et al,^10^ in a meta-analysis evaluating statin-treated patients, concluded that elevated non–HDL-C imparted just as much high risk for cardiovascular events as elevated LDL-C. With the growing evidence that non–HDL-C is a better predictor of outcomes, the American College of Cardiology Foundation and the American Diabetes Association Consensus Conference, in 2008 endorsed non–HDL-C as a cotarget in patients with cardiometabolic risk.^11^

Because statins are considered the first line of therapy, especially for high-risk patients,^2^ our study focused on statin-treated high-risk patients. The first objective of this study was to establish the prevalence of dyslipidemia, especially elevated LDL-C, but also low HDL-C and high TG, in high-risk patients treated with statin monotherapy for >90 days, across 3 “real-world” data sources: electronic medical records (EMR), an administrative claims database, and the National Health and Nutrition Examination Survey (NHANES). Additionally, we assessed patients with only CHD separately from high-risk patients. The second objective was to evaluate the percentages of these high-risk patients achieving ATP III–recommended LDL-C and non–HDL-C goals.

## Methods

This study was a retrospective, cross-sectional, observational study conducted by using 3 different data sources: EMR (GE Centricity), administrative claims data (Clinformatics DataMart, a product of OptumInsight Life Sciences), and a national public health survey (NHANES). This study was conducted by including primarily high-risk patients—that is, patients with previous history of CVD events and CHD or CHD risk equivalents. As per NCEP ATP III,^2^ the LDL-C goal for these patients is <100 mg/dL (Table 1). We also evaluated these high-risk patients for the optional goal of LDL-C <70 mg/dL, as per the 2004 update to the NCEP ATP III Guidelines.^3^ The HDL-C goal was set at ≥40 mg/dL for men and ≥50 mg/dL for women, and the optimal value for TG was <200 mg/dL (Table 1). Finally, non–HDL-C values were calculated as the difference between total cholesterol and HDL-C.^2^ The proportion of patients achieving non–HDL-C goals by LDL-C goal attainment was evaluated. In claims data and EMR data sources, not all patients had total cholesterol values reported. Hence, non–HDL-C values were not evaluated for all patients.

**Table 1.** Recommended Lipid Levels^2,3^

LDL-C goal

<100 mg/dL in the presence of CHD or CHD risk equivalents

<70 mg/dL: Optional goal

Optimal HDL-C

HDL-C ≥40 mg/dL for men

HDL-C ≥50 mg/dL for women

Optimal threshold for TG

<200 mg/dL

Non–HDL-C

Non–HDL-C = Total cholesterol − HDL-C

Non–HDL-C goal <(30 mg/dL + LDL-C goal)

If LDL-C goal was <100 mg/dL, non–HDL-C goal <130 mg/dL

If LDL-C goal was <70 mg/dL, non–HDL-C goal was <100 mg/dL

LDL-C indicates low-density lipoprotein cholesterol; CHD, coronary heart disease; and HDL-C, high-density lipoprotein cholesterol.

**Figure 1.**
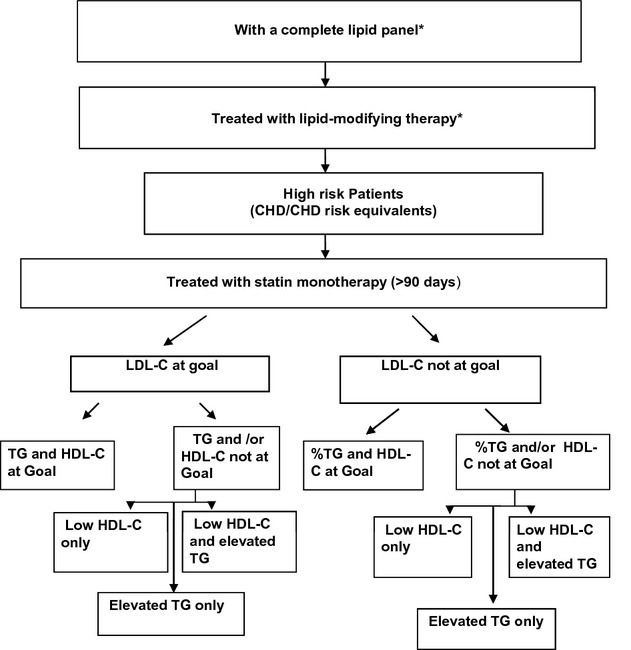
Sample selection for the 3 data sources. *For EMR, our data included only patients who were treated with lipid-modifying therapy; the next step was to identify patients with a complete lipid panel. LDL-C indicates low-density lipoprotein cholesterol; CHD, coronary heart disease; HDL-C, high-density lipoprotein cholesterol; and TG, triglycerides.

Figure 1 provides an overview of the patient selection in all 3 data sources. All high-risk patients from each data source treated with statin monotherapy for >90 days at the time of the lipid panel were identified (Figure 1). The definition of these high-risk patients and definitions of some of the variables differed by data source.

For each of the data sources, as part of a subanalysis, we identified very high-risk patients, including those with CVD (CHD or CHD risk equivalents) plus metabolic syndrome and those with CVD plus diabetes. Metabolic syndrome, while for NHANES was easily evaluated, for the EMR and claims data, was identified on the basis of the presence of ≥3 of the following: low HDL-C (<40 mg/dL for men, <50 mg/dL for women), elevated TG (≥150 mg/dL), diabetes, and hypertension.^2^ The data sources and specific variable definitions are described below.

### Data Sources

#### National Health and Nutrition Examination Survey

NHANES, conducted by the Centers for Disease Control and Prevention, is a program of studies designed to assess the health and nutritional status of adults and children in the United States. This is done via a multistage, probability-based, cross-sectional design, and data are weighted to represent the US population. NHANES surveys a nationally representative sample of 5000 individuals each year across the country. The survey is unique in that it combines interviews and physical examinations. The NHANES interview includes demographic, socioeconomic, dietary, and health-related questions. The examination component consists of medical, dental, and physiological measurements, as well as laboratory tests administered by highly trained medical personnel. Detailed information about the sampling, data collection, testing, and validation are provided by the Centers for Disease Control and Prevention.^12^

The 2007–2008 NHANES was used for the present study. Individuals ≥18 years of age with a complete lipid panel (LDLC, HDL-C, and TG) were selected for the study. Details on the testing for blood lipids are provided by the Centers for Disease Control and Prevention.^13^

NHANES does not collect information on all CHD and CHD risk equivalents. The CHD and CHD risk equivalents identified in NHANES are CHD, myocardial infarction, angina, stroke, and diabetes. Individuals were classified as having a history of CHD on the basis of answers to the questions about CHD, angina, and myocardial infarction for the CHD-specific analyses.

Individuals were classified as having diabetes if they reported that they had been diagnosed with diabetes or were treated with antidiabetics. Treatment with lipid-modifying therapy was identified and classified (therapeutic class), specifically statin monotherapy. Individuals also were asked how long they had been taking the medications. In an effort to increase sample size, we identified undiagnosed patients with diabetes on the basis of glucose levels. Those without diabetes according to self-report of diagnosis or treatment but with fasting glucose >126 mg/dL or oral glucose tolerance test ≥200 mg/dL were identified.^5^ We also identified another set of high-risk patients not diagnosed with CHD or CHD risk equivalents but with 10-year CHD risk >20% according to the Framingham equation, in an effort to evaluate their lipid goal attainment and to further increase sample size for evaluation.

#### Administrative Claims Data

Clinformatics DataMart, a product of OptumInsight Life Sciences, is an administrative claims database of the medical and pharmacy claims for ≍42 million patients enrolled in a large US managed care plan. The latest complete lipid panel (LDL-C, HDL-C, and TG) during January 2003–September 2010 was identified for individuals ≥18 years of age. This lipid laboratory date was set as the index date. These individuals were enrolled in the plan for at least 1 year before and 1 day after the index date. All claims during the year before index date were evaluated for history of CHD or CHD risk equivalents and treatment with specific classes of medications (antidiabetics and lipid-modifying therapy).

Individuals were classified as high risk on the basis of presence of ≥1 claims with *International Classification of Diseases, Ninth Revision (ICD-9)* codes for CHD (410.xx to 414.xx) or CHD risk equivalent: transient ischemic disease/ stroke (433.xx to 434.xx, 437.1); abdominal aortic aneurysm (441.3 to 4); peripheral arterial/vascular disease (443.9, 38.13, 38.18, 39.25, 39.26, 39.29, 39.50, 39.90, 440.2x to 440.4x); diabetes; and revascularization, including coronary artery bypass grafting (36.1, 36.2, 33510 to 33514, 33516 to 33519, 33521 to 33523, 33533 to 33536), percutaneous transluminal coronary angioplasty (36.01 to 36.06, 36.09, 92982, 92984), and stent insertion (36.06, 36.07, 00.63, 92980, 98981, G0290, G0291). Individuals with diabetes were defined as those with ≥1 medical claim with a diagnosis code for diabetes (250.xx) or ≥1 prescription claim for antidiabetics (eg, alpha-glucosidase inhibitors, amilynomimetics, dipeptididyl peptidase 4 inhibitors, incretin mimetics, biguanides, insulins, meglitinides, sulfonylureas, thiazolidinediones). Lipid-modifying therapy at the time of the lipid panel was recorded, and the length of treatment was calculated.

#### Electronic Medical Records

The EMR database (GE Centricity) used for the study is collected from ≍40 000 clinicians and 20 000 nurse practitioners and physician assistants (12 500 medical doctors in the Medical Quality Improvement Consortium system practicing in medium to large group practices). The majority of the physicians are in primary care (85% family practice, internal medicine, obstetrics/gynecology, pediatrics, plus niche specialties). The data are from the *perspective of the treating physician* and are a record of the patient's activity. The data elements include vital signs, laboratory data, observations, complaints, medications, and demographics. Individuals ≥18 years of age whose most recent complete lipid panel (LDL-C, HDL-C, and TG) occurred between January 2003 and September 2010 and who were flagged as active patients (ie, designated in the database as current patients as of September 2010) were identified for the study.

The high-risk patients were identified in this study on the basis of the presence of ≥1 claims with *ICD-9* codes for CHD (410.xx to 414.xx) or CHD risk equivalent: transient ischemic attack/stroke (433.xx to 434.xx, 437.1), abdominal aortic aneurysm (441.3 to 4), peripheral arterial/vascular disease (443.9, 38.13, 38.18, 39.25, 39.26, 39.29, 39.50, 39.90, 440.2x to 440.4x), and diabetes. Individuals were classified as having diabetes in the presence of the *ICD-9* diagnosis code for diabetes (250.xx) or treatment with antidiabetics. The lipid-modifying therapy at the time of the lipid panel was identified, and length of treatment was calculated.

### Data Analyses

All statistical analyses conducted for this study were descriptive in nature. Means with standard deviations are reported, as appropriate. High-risk patients (ie, those with a history of CHD or CHD risk equivalents who were treated with statin monotherapy for >90 days) were identified and analyzed to estimate the prevalence of elevated non–HDL-C, low HDL-C, and high TG by LDL-C goal attainment. Separate analyses were used to estimate prevalence of low HDL-C and high TG by LDL-C goal attainment for each of those patients with CHD. For NHANES, Stata v9.2 software^14^ was used for all analyses. Appropriate weights and variances were applied to estimate population-level proportions. SPSS v16.0^15^ was used to analyze the EMR and claims data.

## Results

In all 3 data sources, 43% to 51% of the high-risk patients treated with statin monotherapy for >90 days had only diabetes, with no other CHD or CHD risk equivalents reported. Using NHANES 2007–2008, a projected US population was identified, consisting of 218.4 million adults ≥18 years of age with a measured lipid panel. In the evaluated population, 16.3% of the US population (projected 35.5 million adults) are treated with some kind of lipid-modifying therapy, and among these, 49.3% (projected 17.5 million) are high-risk patients (CHD or CHD risk equivalents). Among these treated high-risk patients, 59.4% (projected 10.4 million adults) had been treated with statin monotherapy for >90 days at the time of the lipid panel (Table 2). A greater proportion of these high-risk patients treated with statin monotherapy for >90 days had LDL-C <100 mg/dL (76.8%) versus LDL-C <70 mg/dL (24.0%). For both goals of LDL-C <100 mg/dL and LDL-C <70 mg/dL, comparing those who achieved LDL-C goals or had elevated LDL-C, 37.8% to 46.0% had low HDL-C and/or elevated TG.

**Table 2.** Prevalence of Low HDL-C or Elevated TG by LDL-C Goal Attainment (<100 mg/dL, <70 mg/dL) Among High-Risk Patients Treated With Statin Monotherapy for >90 Days in Data Sources

	LDL-C Goal Attainment	n (% of Total)	Low HDL-C Only, n (% of Total)	Elevated TG Only, n (% of Total)	Low HDL-C and Elevated TG, n (% of Total)	HDL-C at Goal and TG <200 mg/dL, n (% of Total)
LDL-C <100 mg/dL						

NHANES	At goal	7.99 MM (76.8)	2.08 MM (20.0)	0.44 MM (4.2)	0.64 MM (6.2)	4.83 MM (46.4)

	Not at goal	2.41 MM (23.2)	0.46 MM (4.4)	0.28 MM (2.7)	0.24 MM (2.3)	1.43 MM (13.8)

Administrative claims data	At goal	86 602 (69.0)	23 921 (19.1)	5590 (4.5)	8883 (7.1)	48 208 (38.4)

	Not at goal	38 871 (31.0)	8466 (6.7)	3782 (3.0)	4301 (3.4)	22 322 (17.8)

EMR	At goal	167 064 (66.6)	60 582 (34.2)	6762 (2.7)	20 848 (8.3)	78 872 (31.5)

	Not at goal	83 636 (33.4)	26 132 (10.4)	6067 (2.4)	12 941 (5.2)	38 496 (15.4)

LDL-C <70 mg/dL						

NHANES	At goal	2.5 MM (24.0)	0.59 MM (5.7)	0.19 MM (1.8)	0.37 MM (3.6)	1.35 MM (13.0)

	Not at goal	7.88 MM (76.0)	1.95 MM (18.8)	0.53 MM (5.1)	0.506 MM (4.9)	4.9 MM (47.2)

Administrative claims data	At goal	25 258 (20.1)	7545 (6.0)	1563 (1.2)	3148 (2.5)	13 000 (10.4)

	Not at goal	100 217 (79.9)	24 842 (19.8)	7809 (6.2)	10 036 (8.0)	57 530 (45.9)

EMR	At goal	65 262 (26.0)	24 658 (9.8)	2623 (1.0)	10 073 (4.0)	27 908 (11.1)

	Not at goal	185 438 (74.0)	62 056 (24.8)	10 206 (4.1)	23 716 (9.5)	89 460 (35.7)

MM refers to “Millions”; the columns “Low HDL-C only,” “Elevated TG only,” “Low HDL-C and elevated TG,” and “HDL-C at goal and TG <200 mg/dL” are mutually exclusive. Please refer to Table 1 and Figure 1 for details. LDL-C indicates low-density lipoprotein cholesterol; HDL-C, high-density lipoprotein cholesterol; TG, triglycerides; NHANES, the National Health and Nutrition Examination Survey; and EMR, electronic medical records.

Because of data availability, we were able to run some additional analyses with NHANES only. Individuals with 10-year CHD risk based on Framingham risk equation and not included in the sample were relatively few. Adding these individuals to the original study sample did not change the results (LDL-C <100 mg/dL: 77.8%; LDL-C <70 mg/dL: 23.8%). The undiagnosed patients with diabetes identified based on elevated glucose levels (fasting glucose or oral glucose tolerance tests) increased the original sample size slightly, and although the percentage attaining LDL-C <100 mg/dL increased slightly from 76.8% to 80.9%, the percentage attaining LDL-C <70 mg/dL did not change. Identifying very high-risk patients, defined as those with CVD and with metabolic syndrome or diabetes, 24.6% and 37.0%, respectively, had LDL-C <70 mg/dL.

A total of 2 615 640 individuals were identified in the administrative claims database who had a complete lipid panel between January 2003 and September 2010, were ≥18 years of age, and were enrolled for ≥1 year before the index lipid panel. A total of 535 831 (20.5%) individuals were being treated with some lipid-modifying therapy, and of these, high-risk patients or individuals with CHD or CHD risk equivalents represented 43.6% (n=233 809). A final sample of 125 473 high-risk patients treated with statin monotherapy for >90 days was identified. Similar to the results from NHANES, the majority of the patients achieved the LDL-C goal of <100 mg/dL (86 602; 69.0%), and only 20.1% (25 258) achieved LDL-C <70 mg/dL (Table 2). Almost half of these high-risk patients (42.6% to 48.5%) treated with statin monotherapy (>90 days) had low HDL-C and/or elevated TG, irrespective of LDL-C goal of <100 mg/dL or <70 mg/dL. When the very high-risk patients were evaluated, including those with CHD or CHD risk equivalents (but not including diabetes as a criterion) along with metabolic syndrome (22.5%) or diabetes (25.9%), the proportion achieving LDL-C <70 mg/dL increased slightly from the original sample data analysis (20.1%).

From the EMR data source, a total of 699 136 patients treated with a lipid-modifying therapy and a complete lipid panel were identified, of whom 359 681 (51.4%) were high-risk patients (CHD or CHD risk equivalent). Among these treated high-risk patients, 250 700 (69.7%) were treated with statin monotherapy for >90 days. Although a majority of the patients achieved the LDL-C goal of <100 mg/dL (167 064; 66.6%), only 26.0% (n=65 262) achieved the LDL-C goal of <70 mg/dL (Table 2). Irrespective of LDL-C goal of <100 mg/dL or <70 mg/dL, among those who achieved LDL-C goal versus not, about half the patients had low HDL-C and/or elevated TG (51.8% to 57.2%). Among the very high-risk patients, defined as those with CHD or CHD risk equivalents (not including diabetes as a criterion) along with metabolic syndrome (32.3%) or diabetes (34.4%), a slightly higher percentage achieved LDL-C <70 mg/dL.

Table 3 provides the demographics of patients selected for the study by LDL-C goal (<100 mg/dL and <70 mg/dL) attainment. The high-risk patients achieving LDL-C goals (<100 mg/dL, <70 mg/dL) were slightly older than their counterparts who did not achieve LDL-C goals in both NHANES and EMR. The claims data had a slightly younger population (56 to 58 years) than the populations of EMR and NHANES.

**Table 3.** Demographics of High-Risk Patients Treated With Statin Monotherapy for >90 Days in Data Sources by LDL-C Goals

	LDL-C Goal <100 mg/dL		LDL-C Goal <70 mg/dL	
		
	At Goal	Not at Goal	At Goal	Not at Goal
NHANES				

Average age (years)±SE	67±1.4	65±1.8	70±1.6	65±1.6

% Men	60.1	52.1	51.3	60.4

% Women	39.9	47.9	48.7	39.6

Administrative claims data				

Average age (years)±SD	56±8.9	57±8.6	58±8.7	57±8.8

% Men	57.3	63.2	65.7	60.2

% Women	42.7	36.8	34.3	39.8

EMR				

Average age (years)±SD	67±11.3	62±12.6	67±11.2	65±12.1

% Men	53.1	44.0	56.1	47.9

% Women	46.9	56.0	43.9	52.1

LDL-C indicates low-density lipoprotein cholesterol; NHANES, the National Health and Nutrition Examination Survey; and EMR, electronic medical records.

To understand potential factors contributing to our results, we evaluated ethnicity and insurance in NHANES and EMR with regards to LDL-C goal attainment but were unable to run the same analyses in the administrative claims data due to the unavailability of ethnicity information and because most of the enrollees had private insurance. In NHANES, only 6.6% had no insurance, and the majority (49.9%) had Medicare. Overall, among those with no insurance, 41.2% had LDL-C <100 mg/dL, and only 10.8% had LDL-C <70 mg/dL. Among those with insurance, more than half had LDL-C <100 mg/dL, and 15% to 35% had LDL-C <70 mg/dL (private insurance [16.3%] and all public insurance [>25%]). In EMR, although only 2% were reported as self-paying, insurance was unknown for 41.5% of the sample. Among those who were self-paying, 48.9% and 18.3%, achieved LDL-C <100 mg/dL and LDL-C <70 mg/dL, respectively. In other groups, 55% to 75% had LDL-C <100 mg/dL, and 23% to 30% had LDL-C <70 mg/dL. In terms of ethnicity, although there were no clear patterns, the majority of patients with LDL-C at goal were white (70% in NHANES and 40% in EMR), and 8% to 9% were African Americans; however, 40% to 50% in the EMR had no ethnicity recorded.

The update for NCEP with the recommendation for the optional LDL-C goal of <70 mg/dL was released in 2004. In EMR and administrative claims data sources, we identified those with a lipid panel between January 2003 and September 2010. Given that at the time of some the lipid panels the updated guidelines were not yet available, and because one of our inclusion criteria was data available during the year before lipid panel, we excluded those with a lipid panel before 2006. Those excluded represented 7% and 30.7% in the EMR and administrative claims samples, respectively. Excluding these patients did not meaningfully change the results. However, in terms of trends, among those with index lipid panel in 2003–2005, a lower percentage of individuals attained LDL-C <100 mg/dL (51.3% in EMR, 63.5% administrative claims data) compared with those whose index lipid panels occurred in the later years (67.8% in EMR, 71.5% administrative claims data). The proportion of patients attaining LDL-C <70 mg/dL with index lipid panel between January 2006 and September 2010 (26.7% in EMR, 21.9% administrative claims data) increased slightly from those with a lipid panel in 2003–2005 (17.9% in EMR, 16.1% administrative claims data).

**Figure 2.**
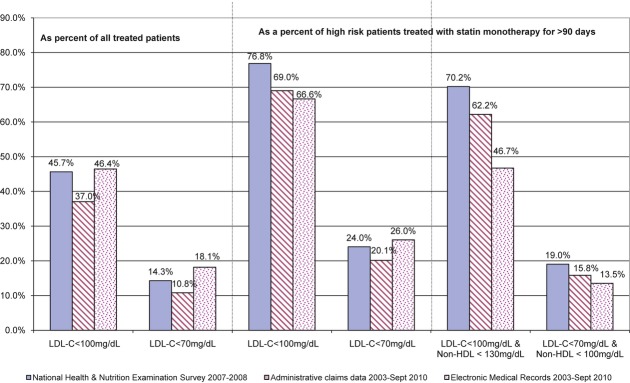
LDL-C goal attainment of high-risk patients treated with statin monotherapy for >90 days. LDL-C indicates low-density lipoprotein cholesterol and HDL, high-density lipoprotein.

Figure 2, comparing goal attainment across the 3 databases, shows that a total of 66.7% to 76.8% of high-risk patients treated with statin monotherapy for >90 days achieved the LDL-C goal of <100 mg/dL, whereas only 20.1% to 26.0% achieved LDL-C <70 mg/dL. These patients achieving LDL-C <100 mg/dL and <70 mg/dL account for 37.0% to 46.4% and 10.8% to 18.1%, respectively, of all treated (any lipid-modifying therapy) patients. In terms of achieving both LDL-C and non–HDL-C goals, 46.7% to 70.2% of high-risk patients treated with statin monotherapy for >90 days had LDL-C <100 mg/dL and non–HDL-C <130 mg/dL. However, a much smaller proportion of these statin-treated high-risk patients (13.5% to 19.0%) had LDL-C <70 mg/dL and non–HDL-C <100 mg/dL.

**Figure 3.**
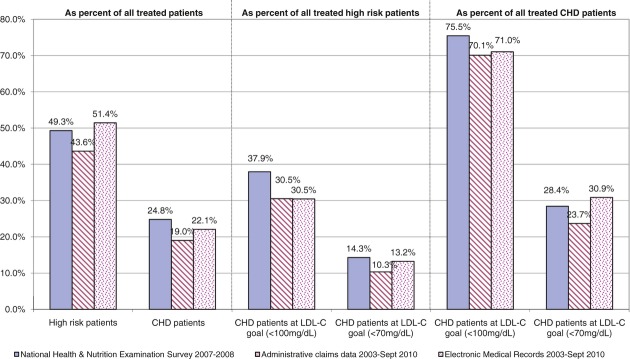
Treated CHD patients achieving LDL-C <100 mg/dL and <70 mg/dL in 3 data sources. CHD indicates coronary heart disease and LDL-C, low-density lipoprotein cholesterol.

Evaluating only the treated CHD patients in all 3 data sources, Figure 3 reports percentages of all treated CHD patients achieving LDL-C <100 mg/dL and <70 mg/dL. In the EMR data, among CHD patients treated with some lipid-modifying therapy, 71.0% of patients had LDL-C levels <100 mg/dL, and only 30.9% achieved LDL-C <70 mg/dL. Similarly, in claims data and NHANES, 70.1% and 75.5% of treated CHD patients achieved LDL-C <100 mg/dL, and 23.7% and 28.4% achieved LDL-C <70 mg/dL, respectively.

## Discussion

Our study is the first of its kind evaluating 3 diverse contemporary databases with regards to lipid goals in statin-treated subjects. In our analysis of 3 “real-world” databases, in high-risk patients treated with statin monotherapy for >90 days, 67% to 77% achieved LDL-C <100 mg/dL, and 20% to 26% achieved the optional goal of LDL-C <70 mg/dL (Figure 2). When we evaluated high-risk patients treated for >180 days with statin monotherapy, similar results were seen in the proportion of patients attaining LDL-C goals (<100 mg/dL, <70 mg/dL). Additionally, comparing those who achieved both LDL-C goals versus those who did not, we found that between 40% and 60% of them also had low HDL-C and/or elevated TG. With the very high-risk patients (CVD patients with metabolic syndrome or diabetes), only a third of them (20% to 35%) in both the EMR and the administrative claims data achieved a LDL-C <70 mg/dL. Our results are similar to others in the literature that show a temporal improvement in attaining the high-risk LDL-C goal of <100 mg/dL, but they also highlight the challenge of achieving the optional <70 mg/dL goal, which many lipid experts believe should no longer be optional in individuals with established vascular disease.

Other published studies have examined the NHANES data for the percentage of at-risk population attaining their recommended LDL-C goal. Gandehari et al^16^ analyzed data from the 2003–2004 NHANES and found that only 36% to 37% of those with CVD or related comorbidities were at recommended levels for LDL-C and non–HDL-C. The low proportion of patients achieving LDL-C <100 mg/dL in that study is probably due to the use of data for the entire population, unlike our study, which focused on high-risk patients.

The proportion of high-risk patients achieving optional LDL-C <70 mg/dL in the published literature is more comparable to the present study than the proportion of patients achieving LDL-C <100 mg/dL. Several epidemiological studies, including Return on Expenditure Achieved for Lipid Therapy in Asia (REALITY-ASIA), INTERHEART, Lipid Treatment Assessment Project (LTAP)-1, LTAP-2, and National Cholesterol Education Program Evaluation Project Utilizing Novel E-technology (NEPTUNE) II, which evaluated high-risk patients treated with statins or some other lipid-modifying therapy, reported a wide variation (18% to 43%) in attainment of LDL-C <100 mg/dL, depending on the definition of high-risk patients (CHD versus diabetes) and race.^17–23^ However, some studies have reported a high proportion of patients (50% to 70%)^24–28^ attaining LDL-C levels <100 mg/dL, similar to the present study. Also, among the very high-risk patients, 15% to 30%^25–28^ are reported to achieve the optional goal of LDL-C <70 mg/dL.

In terms of trends, in the present study, a lower percentage of individuals with an index lipid panel in 2003–2005 attained LDL-C <100 mg/dL (51.3% in EMR, 63.5% administrative claims data) than those with an index lipid panel in the later years (67.8% in EMR, 71.5% administrative claims data). This improvement in goal attainment over the years has been reported by LTAP-1^19^ and LTAP-2^24,25^ and by Cohen et al,^29^ who evaluated LDL-C goal attainment in NHANES over time. Cohen et al^29^ examined trends across NHANES surveys and found that there was a progressive increase in percentage of the population achieving LDL-C <100 mg/dL, from 17% in NHANES II (1976–1980), to 23% in NHANES III (1988–1994), to 31% in NHANES (1999–2006). Similarly, Kuznik and Mardekian^30^ evaluated the percentage of the population with diabetes reaching LDL <100 mg/dL in NHANES 1999–2008 and reported that the proportion of treated patients reaching that goal increased from about 30% in 1999–2000 to 54% in 2007–2008. Most of the increase in goal attainment can be traced to higher rates of adherence to statin treatment.

In the EMR data source, the majority of those with LDL-C not at goal were women. This might be due to the data source itself or to regional differences, because some areas were overrepresented. Evaluating insurance coverage and ethnicity to understand the potential factors influencing our results did not yield any definite trends. In general, fewer individuals with no insurance than individuals with some form of insurance had lipid levels at goal. However, in the EMR data, ≍40% had no insurance information. Similarly, evaluation of ethnicity in both NHANES and EMR showed that the majority of individuals at LDL-C goal were white, followed by African Americans.

In the present study, 46% to 70% achieved both LDL-C <100 mg/dL and non–HDL-C <130 mg/dL, with 13% to 19% achieving LDL-C <70 mg/dL and non–HDL-C <100 mg/dL. This is better than NEPTUNE II,^26^ which reported that only 27% of the patients attained both LDL-C (<100 mg/dL) and non–HDL-C goals. Recently, in a study among CHD patients in a Veterans Affairs hospital network, 51% had both LDL-C (<100 mg/dL) and non–HDL-C at goal, and 13% had both the optional LDL-C <70 mg/dL and non–HDL-C at goal.^31^

### Limitations

This was an observational cross-sectional study using secondary data sources, namely the EMR and claims data, and NHANES is a self-report survey with available laboratory tests and examination data. Given the limitations of the data sources listed in this section, we have tried to address as many of them as possible. These data sources do not provide any information on the adherence to medication, nor do they document dietary or exercise habits that might have been used to adjunctively control lipid levels. In addition, these data sources do not have detailed clinical information and notes that could have been used to make treatment decisions. Numerous sociodemographic, cultural, and other factors are beyond the scope of this study. Because this is a cross-sectional study, it does not take into account the variability of lipid values over time. There might or might not be overlap in patients from EMR and the administrative claims data. Because both are de-identified, there is no way for us to check this overlap.

EMR documents the intent of the physician to prescribe but not necessarily what was dispensed at the pharmacy. The EMR has limited records that include procedure codes (such as stents, coronary artery bypass grafting, percutaneous transluminal coronary angioplasty), and therefore they were not included in the definitions of some CHD and CHD risk equivalents. In the claims data and EMR, it is unknown if the TG measurements were nonfasting. Although the claims data include a record of a patient filling a prescription, we are unable to ascertain if the patient took the medication. Not all enrollees in the administrative claims data or all patients in the EMR had a complete lipid value, and some did not have any lipid value.

In both EMR and claims data sources, *ICD-9-CM* codes were used to identify CHD and CHD risk equivalents. Given that diagnosis codes can have overcoding and undercoding issues, it is essential to conduct data quality checks, run sensitivity analyses as appropriate, and compare results to literature if available.^32^

The initial vague symptoms of angina and the need for rigorous testing for confirmed diagnoses can lead to erroneous diagnosis coding for angina. To address this, we redid all analyses with angina excluded from the definition of CHD and CHD risk equivalents. The results from these sensitivity analyses did not change our original results. Comparing the results across data sets as well as with literature, we found very consistent results, which indicate that bias in the coding in this study was minimal.

In NHANES, information on all cardiovascular and other comorbidities is not collected. Moreover, these comorbidities are self-reported and are subject to biases such as low reporting due to lack of awareness. Because diabetes is one of the self-reported CHD / CHD risk equivalents, we also used treatment for diabetes to improve accuracy of the estimates. In addition, we identified those with elevated glucose (fasting glucose test or oral glucose tolerance test). Addition of these unreported individuals with diabetes did not change our results. The only CHD / CHD risk equivalent information that NHANES collects is presence of CHD, angina, myocardial infarction, stroke, or diabetes. Other CHD risk equivalents, including symptomatic carotid artery disease, peripheral arterial disease, and abdominal aortic aneurysm, were not collected in NHANES. Results from NHANES analyses are a projection and are dependent on weights and other adjustments made for sampling, although these are well characterized and validated.

The 2004 update for ATP III recommended the optional LDL-C goal of <70 mg/dL. In EMR and administrative claims data sources, we identified those with a lipid panel between January 2003 and September 2010. Given that the updated guidelines were not yet available at the time of some of the lipid panels and that one of our inclusion criteria was data available during the year before lipid panel, we excluded those with a lipid panel before 2006, which represented 7% and 30.7% in EMR and in the administrative claims sample, respectively. Excluding these patients did not change our original results.

### Looking Forward

Some of the reasons for a wide range in the proportion of high-risk patients achieving the LDL-C goals could be related to study design (cross-sectional versus cohort studies) or the definition of high risk. On the other hand, there could be other reasons, including system barriers (e.g., referrals and cost and access to care) and patient-related issues (e.g., adherence and comorbidity burden). Although cost is a significant barrier, the literature supports the fact that it is not the main barrier to access to or adherence to medication.^33^ Consistent evidence of low adherence to lipid-modifying medications emerges as a plausible reason for suboptimal lipid goal attainment.^34^ The reasons for low adherence to life-saving lipid-modifying cardiovascular medication, especially among individuals with established vascular disease, need more attention and solutions because the cost-effectiveness of increasing adherence seems substantial.^35^

## Conclusion

Our evaluation of NCEP ATP III LDL-C and non–HDL-C goal attainment across 3 diverse databases shows that there is significant room for improvement. Our results are consistent with the results reported in the literature for the United States and for other international databases and surveys. All providers and payers in the healthcare system need to identify and neutralize the barriers to medication adherence and implement new methods to incentivize adherence to statins and other CVD medications in high-risk patients.
